# Physical Activity Impacts of an Activity-Friendly Community: A Natural Experiment Study Protocol

**DOI:** 10.3389/fpubh.2022.929331

**Published:** 2022-06-15

**Authors:** Xuemei Zhu, Marcia G. Ory, Minjie Xu, Samuel D. Towne, Zhipeng Lu, Tracy Hammond, Huiyan Sang, J. Timothy Lightfoot, E. Lisako J. McKyer, Hanwool Lee, Ledric D. Sherman, Chanam Lee

**Affiliations:** ^1^Department of Architecture, Texas A&M University, College Station, TX, United States; ^2^Center for Health Systems & Design, Texas A&M University, College Station, TX, United States; ^3^Department of Environmental and Occupational Health, School of Public Health, Texas A&M University, College Station, TX, United States; ^4^Center for Population Health and Aging, Texas A&M University, College Station, TX, United States; ^5^Department of Landscape Architecture and Urban Planning, Texas A&M University, College Station, TX, United States; ^6^School of Global Health Management and Informatics, University of Central Florida, Orlando, FL, United States; ^7^Disability, Aging, and Technology Cluster, University of Central Florida, Orlando, FL, United States; ^8^Southwest Rural Health Research Center, Texas A&M University, College Station, TX, United States; ^9^Department of Computer Science & Engineering, Texas A&M University, College Station, TX, United States; ^10^Department of Statistics, Texas A&M University, College Station, TX, United States; ^11^Department of Health and Kinesiology, Texas A&M University, College Station, TX, United States; ^12^Center for Community Health Development, Texas A&M University, College Station, TX, United States

**Keywords:** physical activity, obesity, activity-friendly community, natural experiment, healthy aging, healthy community, active living, study protocol

## Abstract

**Background:**

Stakeholders from multiple sectors are increasingly aware of the critical need for identifying sustainable interventions that promote healthy lifestyle behaviors. Activity-friendly communities (AFCs) have been known to provide opportunities for engaging in physical activity (PA) across the life course, which is a key to healthy living and healthy aging.

**Purpose:**

Our purpose is to describe the study protocol developed for a research project that examines: (a) the short- and long-term changes in total levels and spatial and temporal patterns of PA after individuals move from non-AFCs to an AFC; and (b) what built and natural environmental factors lead to changes in PA resulting from such a move, either directly or indirectly (e.g., by affecting psychosocial factors related to PA).

**Methods:**

This protocol is for a longitudinal, case-comparison study utilizing a unique natural experiment opportunity in Austin, Texas, USA. Case participants were those adults who moved from non-AFCs to an AFC. Matching comparison participants were residents from similar non-AFCs who did not move during the study period. Recruitment venues included local businesses, social and print media, community events, and individual referrals. Objectively measured moderate-to-vigorous PA and associated spatial and temporal patterns served as the key outcomes of interest. Independent (e.g., physical environments), confounding (e.g., demographic factors), and mediating variables (e.g., psychosocial factors) were captured using a combination of objective (e.g., GIS, GPS, Tanita scale) and subjective measures (e.g., survey, travel diary). Statistical analyses will be conducted using multiple methods, including difference-in-differences models, repeated-measures linear mixed models, hierarchical marked space-time Poisson point pattern analysis, and hierarchical linear mixed models.

**Conclusion:**

Natural experiment studies help investigate causal relationships between health and place. However, multiple challenges associated with participant recruitment, extensive and extended data collection activities, and unpredictable intervention schedules have discouraged many researchers from implementing such studies in community-based populations. This detailed study protocol will inform the execution of future studies to explore how AFCs impact population health across the life course.

## Introduction

Policy-relevant and sustainable health-promoting interventions have the potential to improve population health and support healthy living across the life course leading to healthy aging. However, it is not always feasible to conduct experimental studies on such interventions (e.g., policy changes, environmental modifications), especially when the intervention involves large-scale changes in community environments (e.g., residential relocation). Natural experiments allow researchers to overcome such feasibility challenges and better understand the effectiveness of such interventions using advanced research designs and methodological approaches that aim to strengthen cases for causal inference (1, 2). This paper presents the study protocols used to execute a natural experiment that assessed a policy-relevant and health-promoting intervention, the implementation of an activity-friendly community (AFC) design, to assess its impact on residents' physical activity (PA).

Obesity is a growing public health problem globally, with nearly a third of the world population being overweight or obese (3). In the USA, obesity has increased even more dramatically, now reaching over 40% of American adults (4). Obesity is a major risk factor for the onset or exacerbation of many chronic conditions such as heart diseases, diabetes, and cancer (5, 6). PA can help combat the obesity epidemic and brings many other health benefits (7). Guidelines from the US Centers for Disease Control and Prevention (CDC) recommend most adults engage in at least 150 min of moderate-intensity or 75 min of vigorous-intensity aerobic PA per week, or a combined equivalent to at least 150 min of moderate-to-vigorous physical activity (MVPA) per week (7). Staying physically active throughout the life course is also a key factor for healthy aging (8). However, in 2018, 46% of American adults did not meet these PA guidelines (9), and older adults (65+ years of age) are among the least likely to meet PA guidelines with less than a quarter meeting the recommendation as of 2019 (10, 11).

There has been a recent paradigm shift from individual-focused behavior change models to ecological models that consider the complex system of personal, social, and physical environmental factors that affect one's decision and ability to be physically active (12–16). Moderate and utilitarian/lifestyle PA (e.g., walking for transportation) integrated into the daily routine is often more attractive, sustainable, and cost-effective than purely recreational or structured PA, especially among those at high risk for obesity (16–19). That stated, lifestyle PA requires supportive living environments for viability. This is especially true for children and older adults and people with limited resources to access other PA amenities (e.g., paid gym membership) (16, 20).

The built and natural environments of residential communities have become increasingly recognized as important venues for promoting PA at the population level (20, 21). Living in AFCs with mixed and compact land uses, well-connected street networks, complete pedestrian and bicycle infrastructure, diverse recreational facilities, and slow/managed traffic has been associated with increased PA among adults (15, 17, 21–27). Such communities bring everyday destinations and homes closer to each other and make physically active travel modes (e.g., walking) viable and attractive. The natural environmental features of the neighborhood such as parks, lakes, trails, trees along streets, and visually appealing natural scenery, have also been linked with health benefits such as increased PA, reduced stress and depression, and improved overall well-being (12, 23, 28–31).

Personal attitudes (e.g., personal beliefs, self-efficacy, and perceived barriers) and social influences related to social support, social capital, safety, and social norms, have also been linked to PA (32–39). Social support for PA (e.g., having someone to exercise with) has been reported to be the most clearly established interpersonal determinant of PA (36–39). Limited studies have also suggested that AFCs facilitate social interactions among neighbors and help increase a sense of community (40–44). Perceived safety is recognized as an important barrier to PA, with safety concerns constraining PA (35, 45). More recent ecological models are positing a host of psychosocial factors as mediators between the physical environment and PA (32, 46–48).

Despite the substantial body of evidence on the association between the physical environment and PA, most cross-sectional studies do not address the potential self-selection bias (e.g., residents interested in PA intentionally choosing to live in an AFC). A limited number of studies have utilized proxies of self-selection (attitudinal and residential preference variables) and provided promising results supporting the significant roles of the built environment on PA even after accounting for self-selection (49, 50). Moreover, a systematic review identified 23 studies on the effects of residential relocation on PA, walking, and travel behavior (51). The findings were encouraging, especially for the relationship between residential relocation to more activity-friendly locations and increased walking, but somewhat weak among prospective studies or other outcomes (e.g., total PA, cycling, transit use, and driving).

Overall, despite the growing body of literature, most previous studies on environment-PA relationships are cross-sectional, thus limiting the ability to understand complex pathways among multi-level factors and establish causal relationships between environmental interventions and increases in PA (17, 52–56). Also, many studies on PA promotion have focused on the changes in the total amount of PA, but did not explore the “when,” “where,” and “why” of those changes for total and specific types of PA, and the potential short- and long-term PA impacts of environmental interventions (52–55, 57). Another key limitation of some prior studies is the sole reliance on self-reports of PA, which have been shown to have significant measurement errors (58). These methodological limitations and unanswered questions prevent a full understanding of underlying mechanisms about how environmental interventions may promote PA (12, 15, 57).

On the other hand, the demands and market acceptance for AFCs have been growing. Urban planning trends such as New Urbanism, Smart Growth, and the Leadership in Energy and Environmental Design-Neighborhood Development (LEED-ND) advocate AFCs for their benefits not only on health but also on sustainability, economy, and equity (59–61). However, the traditional urban planning and land development process does not fully integrate the health benefits of their practices into the decision-making process. Recently, there have been growing efforts to overcome policy barriers to the development of AFCs. Agencies such as the Environmental Protection Agency and the American Planning Association have identified possible policy solutions such as revisions of local zoning codes (62–64). More research is needed to provide more confirmatory evidence about the health benefits of AFCs, and thereby help inform future environmental/policy interventions and overcome existing regulatory barriers.

To help address the relative gaps in the existing literature identified above and support rigorous environment-PA studies, this paper provides a comprehensive description of the study protocol developed to execute a quasi-experimental study (natural experiment) using a pre-post, case-control design. This approach allows researchers to assess the implementation of an AFC design, and to test causal inference using a difference-in-differences framework (65, 66). We also summarize lessons learned from a transdisciplinary team approach used in this study, which allowed us to formulate research questions and implement the research with insights from multiple disciplines, informed by the diverse methods and approaches from different fields.

## Methods and Analysis

### Study Design and Aims

This paper describes the study protocol for the Active Living Austin (ALA) study, which is funded by the National Institutes of Health (Grant ID: R01CA197761, 2015-2022). ALA investigates a timely and understudied topic—the causal relationships between changes in physical environments and PA, utilizing a natural experiment with residents moving to and living in an AFC (Mueller community in Austin, Texas, USA). It is designed to make several substantive and methodological innovations in studying environmental approaches toward PA promotion across the life course. With a longitudinal, case-comparison study design, ALA aimed to examine the short- and long-term impacts of the residential relocation, while capturing both the total amount and the spatial and temporal patterns (frequency, timing, type, location) of PA. To better understand the complex causal pathways at multiple levels (personal, social, physical environment), ALA examines both the direct and indirect impacts (through the psychosocial mediators) of the residential relocation on PA. A common challenge for these types of real-life studies is the possible self-selection bias because researchers cannot randomly assign participants to different living environments. To assess and reduce the impact of self-selection bias, we also included validated self-selection-related items (e.g., residential preferences, reasons for the household relocation, and attitudes and preferences related to PA) in the survey component of this study.

The specific aims and corresponding hypotheses are drawn from existing literature and practice and summarized below.

#### Aim 1

Examine the short- and long-term changes in total PA levels and spatial and temporal patterns of PA, after individuals move from non-AFCs to an AFC.

##### Hypothesis 1

Compared to the pre-move baseline and comparison participants, case participants will (1A) achieve greater short-term increases in PA levels after moving to the AFC; (1B) maintain increased PA levels over baseline at long-term, post-move follow-ups; and (1C) have a higher proportion out of total PA that takes place within the community, a higher proportion of walking out of total PA, and more PA bouts/sessions throughout the day indicating PA is more integrated into daily routines, at post-move follow-ups.

#### Aim 2

Determine what built and natural environmental factors lead to changes in PA among individuals moving from non-AFCs to an AFC, either directly or indirectly (by affecting psychosocial factors related to PA).

##### Hypothesis 2

Environmental factors in the AFC such as increased density, land use mix, sidewalks, walking/hiking paths, water features, and parks, will lead to increased PA both directly and indirectly (by improving attitudes toward and community support for PA) among case participants, while the absence of such environmental changes for comparison participants (non-movers staying in their non-AFCs) is associated with lack of increases in their PA levels.

### Conceptual Framework

**Figure 1:**
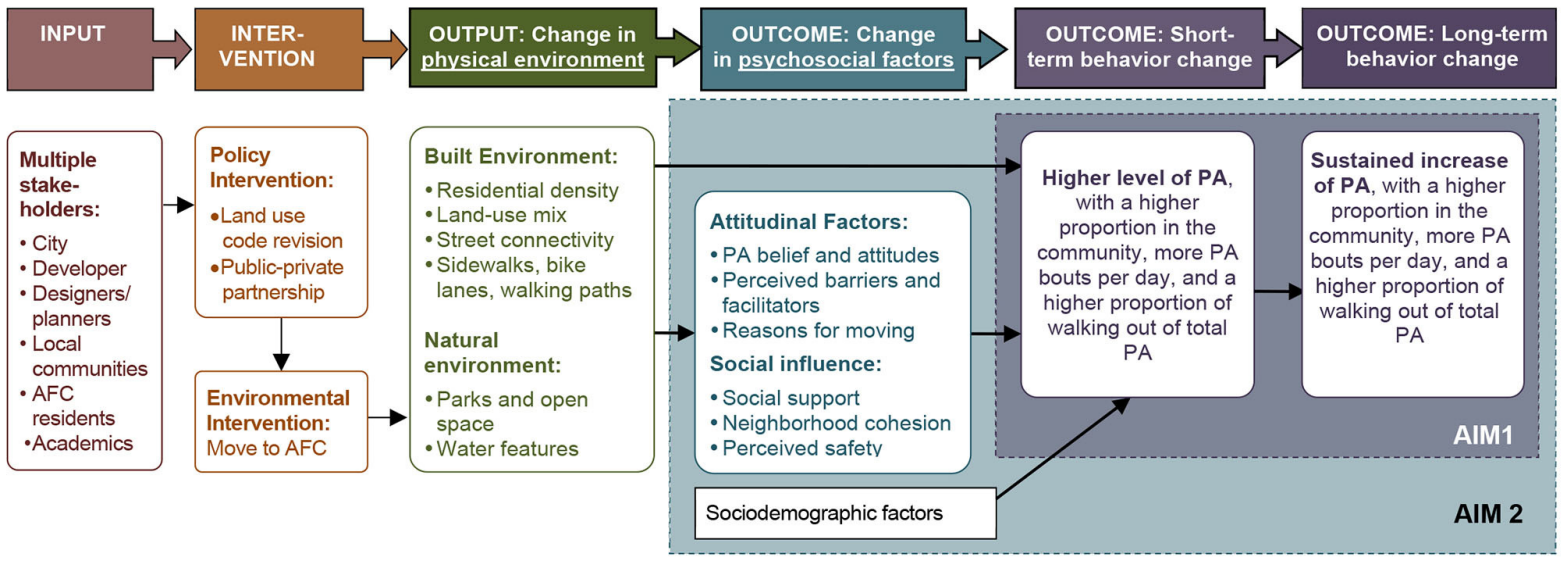
Logic model for the Active Living Austin study.

Drawing on the ecological model as the conceptual basis to understand PA behaviors (54), we developed a logic model (Figure 1) to illustrate the relationships among the resources (input), program activities (interventions), outputs, and short- and long-term outcomes of the intervention (moving to an AFC) (67–70). Psychosocial factors, including personal attitudes and social influences, are the hypothesized mediators between the environmental intervention and PA changes.

### Study Setting: Case and Comparison Communities

**Figure 2:**
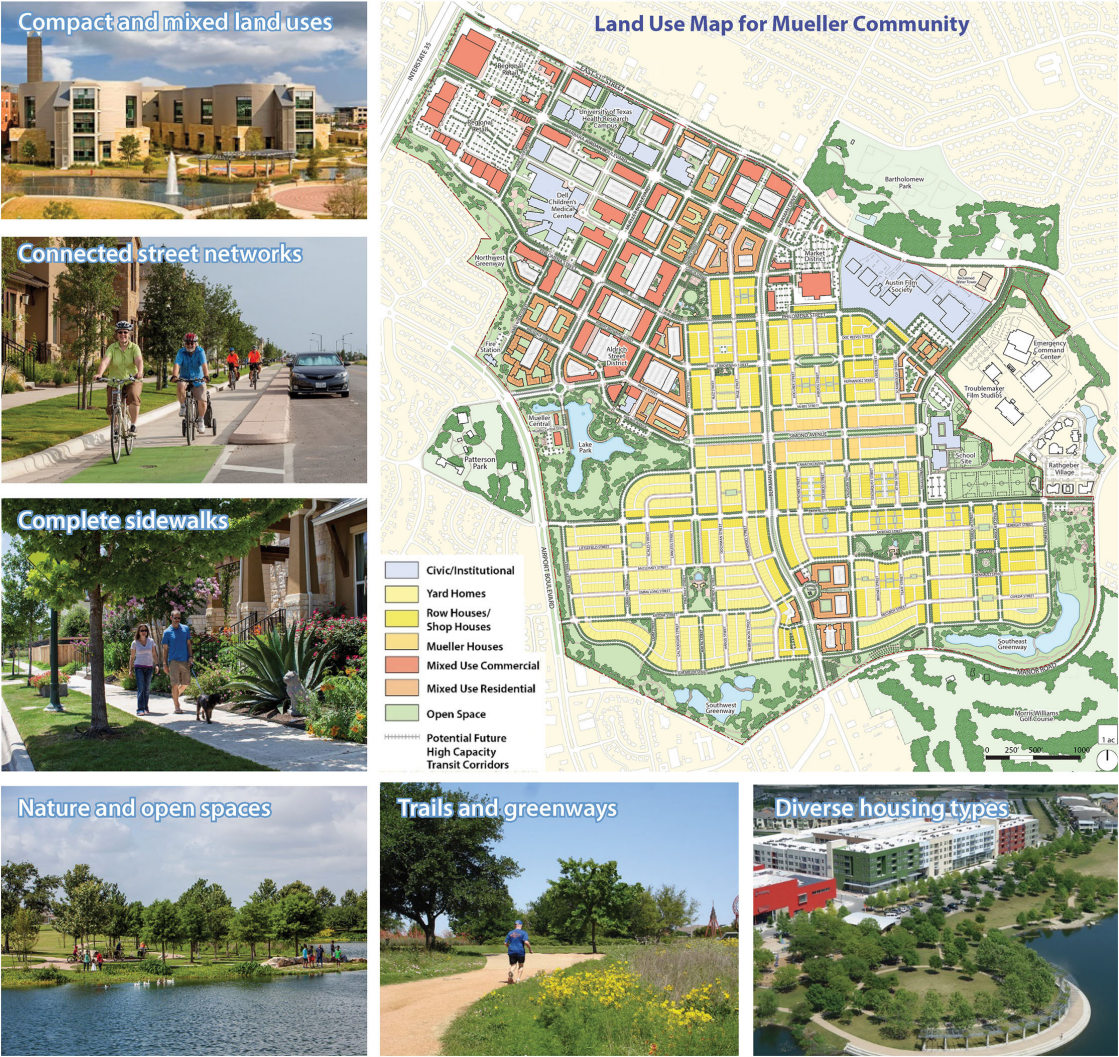
Land use map and photos of the Mueller Community in Austin, Texas, USA. [Source of images: Catellus Development Corporation. Note: An older version of the land use map was published earlier in the journal of World Health Design (71)].

The case community (AFC) is the 700-acre Mueller community in Austin, Texas, USA. It will accommodate about 16,400 residents and 16,000 employees upon completion (estimated to be around the end of 2024). The community has reserved about 25% of the housing units as affordable homes for households with incomes lower than the area's median. Mueller's activity-friendly environment represents a departure from typical community developments in the city. Compact and mixed land uses, well-connected street networks, complete sidewalks, and rich PA amenities (e.g., green/open spaces, trails, and greenways), as well as diverse housing types, are notable features (see Figure 2 and Table 1).

**Table 1:** Physical environment and population characteristics of the activity-friendly community (AFC) (Mueller community) and City of Austin (71)^a^ at the time of the pilot study (2013).

	**Features**	**City of Austin**	**Mueller Community**
**Physical Environment** ^**b**^	Population density (persons/acre)	Mean: 6.8 (SD^c^: 3.7)	14
	Land use mix	Mean: 0.45 (SD: 0.24) (range: 0–1)^d^	10,000 employees, 10,000 residents, and 366,000 square feet of retail space on the 711-acre site
	Street connectivity (intersections/100 acres)	Mean: 19.7 (SD: 11.3)	66
	Sidewalk coverage (%)	Mean: 23.7 (SD: 13.7)	100
	Parks and open space coverage (%)	Mean: 8.9 (SD: 9.6)	20 (Each household has green space within 600 feet.)
**Population** ^**e**^	Hispanic or Latino (of any race)	31.4%	35.1%
	White (one race)	68.3%	71.4%
	Population under the age of 18	22.1%	21.9%
	Mean household income	$68,659	$66,923

a *This table was first published in the journal of World Health Design and republished here with permission (71)*.

b *Physical environmental measures for the City of Austin were based on the authors' previous measures of 74 neighborhoods (defined as public elementary schools' attendance areas) in Austin (72). Physical environmental features of the Mueller community in this table represent the master plan at time of our pilot study (2013). As of May 2022, Mueller has about 750,000 square feet of retails space, and is projected to accommodate about 16,400 residents and 16,000 employees upon completion by the end of 2024*.

c *SD: Standard deviation*.

d *The land-use mix measure describes the evenness of land use distribution based on the square footage of residential, commercial, and office land uses (73). The value ranges from 0 (single land use) to 1 (a perfectly even mix)*.

e *The population information was obtained from the 2010 Census and the 2005-2009 American Community Survey (74)*.

Examining available data during our pilot study (2013) revealed a similarity in Mueller's population characteristics at that time to the citywide average (Table 1) (71). As of February 2014, when we started planning this study, Mueller had approximately one-quarter of its construction completed, with about 4,750 residents living in its 1,900+ homes (859 single-family homes and 1,000+ apartments), and about 4,850 employees working in the community. According to the latest Mueller Community Report from December 2018 (mid-point of our study period), Mueller housed about 8,500 residents in 3,500 housing units and served as a workplace for about 5,500 employees. Comparison communities for this study are non-AFCs where case participants lived before moving to Mueller and other similar non-AFCs in Austin.

### Study Population

Two types of participants were recruited for the study: case and comparison participants. Case participants are those moving from non-AFCs to this AFC and additional inclusion criteria include: (a) being 21+ years of age; (b) not a full-time student; (c) having no physical impairment or disability preventing engagement in PA; and (d) planning to live in this AFC for at least 1 year. Utilizing similar inclusionary criteria, comparison participants were recruited from case participants' pre-move communities and other communities in Austin with similar physical environmental features in terms of lack of support for PA. They were matched with case participants in the data analysis stage using propensity score matching considering covariates such as sex, age, race, ethnicity, income, baseline weekly minutes of MVPA, and Walk Score of the participant's home location.

### Recruitment

Recruitment presented one of the most significant challenges due to the need to recruit case participants before they moved to Mueller for the baseline data collection. We developed a multi-channel recruitment strategy based on our experiences from the pilot study (75). Considering the varying locations from which case participants moved and the unpredictable times of move, we had to maintain a flexible, multi-phased approach, recruiting participants on a rolling basis. Venues for the case participant recruitment included local businesses such as the leasing offices of apartment buildings and the developer's office, home builders, and realtors; online messaging *via* social media, community online newsletters, and project website; print media (e.g., study flyers, local newspaper); individual referral by local residents who knew of someone moving to Mueller; and community venues such as the Mueller Neighborhood Association meetings and local events. Comparison participants were recruited through case participants' referrals, local neighborhood organizations, and media advertisements.

### Study Variables and Data Collection

**Figure 3:**
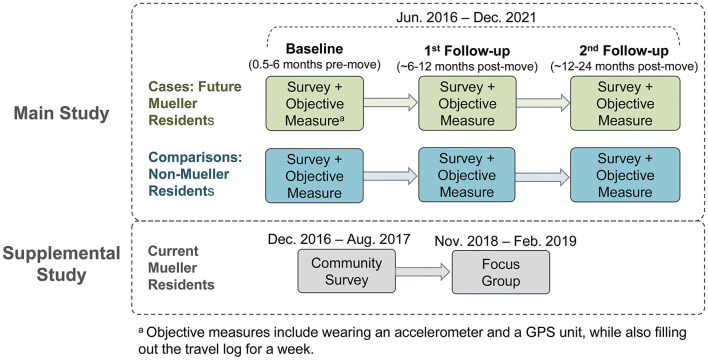
Data collection process of the Active Living Austin study.

Table 2 lists the study variables and their corresponding data sources and measurement methods. One baseline and two follow-up assessments were conducted. For case participants, the baseline was operationally defined as 0.5–6 months before moving to the AFC, and follow-up was operationally defined as short-term (first follow-up at about 6–12 months post-move) and long-term (about 12–24 months post-move) (Figure 3). Follow-up measurements for comparison participants were conducted at about 6–12 months and 12–24 months from their baseline measurement, respectively, to approximate the time intervals for case participants' baseline and follow-up assessments. In addition to this main study with a pre-post, case-control design, a supplemental study—Community Survey of current Mueller residents—was conducted between December 2016 and August 2017 to assess current Mueller residents' PA patterns within and outside Mueller and the perceptions of their neighborhood environment. A follow-up qualitative study was carried out with a subgroup of Community Survey participants to identify factors serving as barriers and facilitators to physical and social health among these Mueller community residents. Data for this qualitative study were collected *via* focus group sessions between November 2018 and February 2019 with the subgroup of residents, who resided in senior living or affordable housing units, or self-identified as members of an ethnic/minority group within the Mueller community.

**Table 2:** Study variables and measurement methods.

		**Variables**	**Measurement details**	**Measurement source**
**Outcome variables**	H^a^-1A; H-1B	Change in total physical activity (PA)	Daily minutes of moderate-to-vigorous PA (MVPA)	Accelerometer: ActiGraph GT3X+ Self-reported PA: Items adapted and/or modified from the International Physical Activity Questionnaire (IPAQ) to measure hours and minutes of MVPA in a typical week
	H-1C	Change in spatial and temporal patterns for specific types of PA	Percentage of MVPA taking place within the 0.25-, 0.5-, and 1-mile street network buffers around home	Accelerometer: ActiGraph GT3X or GT3X GPS: Qstarz BT Q1000 XT Travel log
			Number of PA bouts/sessions per day	
			Percentage of total PA accounted by walking	
**Mediators/ Intermediate outcomes**	H-2	Change in psycho-social factors	*Attitudinal factors:* PA beliefs and attitudes, perceived barriers and facilitators, reasons for moving	Survey: Items adapted and/or modified from the “Healthy Community Survey” (75), which was adapted in part from the “Twin City Walking Study,” the “Active Where Survey,” and the “Neighborhood Environment Walkability Scale (NEWS)” (76–79)
			*Social influence:* social support, neighborhood cohesion, and perceived safety	
**Independent and control variables**	Personal factors		Age, sex, ethnicity, education, health status, marital status	Survey: Items adapted and/or modified from the BRFSS Questionnaire (80)
			Quality of life	Survey: Items adapted and/or modified from the “EQ-5D-5L” (81, 82) and the BRFSS
			Weight, height, body composition	Tanita Scale: TBF-400 Total Body Composition Analyzer Height measure: HM-200P Portstad Stadiometer
	Household factors		Household income, number of children	Survey: Items adapted and/or modified from the BRFSS Questionnaire (80)
	Physical environment		*Built:* Population density (persons/acre), land use mix entropy (0-1), proximity to and density of PA facilities and utilitarian destinations, street connectivity (intersection density), sidewalk completeness, transit stop density, traffic speed	GIS: proximity and buffer (0.5-mile airline, network, and sausage buffer) measures around each respondent's home, using ArcGIS version 10.6; raw GIS data for land use, density, streets, traffic, sidewalks, bike lanes, transit stops, etc. from City of Austin, ESRI, Austin Transit Authority, Capital Area Metropolitan Planning Organization, etc.
			*Natural:* Proximity to, coverage of, and density of parks, trails/paths, water features, and other open spaces; tree canopy (%); mean slope (%)	GIS: Proximity and buffer (0.5-mile airline, network, and sausage buffer) measures around each respondent's home, using ArcGIS version 10.6; raw GIS data from Austin Parks & Recreation Department, Digital Orthophoto Quadrangle (DOQQ) images, and Digital Elevation Model (DEM) data from US Geological Survey.

a *H, Hypothesis*.

#### Measurement of PA Using Accelerometer, GPS, and Travel Log

PA level was captured through a self-report survey (conducted online *via* Qualtrics or using a paper copy) for all participants and objectively through accelerometer and GPS units for the sub-group of participants willing to join the objective measure. Surveys were distributed to all participants *via* email or during the introductory in-person meeting. The survey instrument was composed of standardized items adopted or adapted from published work to ensure both practicality and psychometric quality (Table 2) (75, 76, 78–84), and was designed to be completed in ~20–30 min. In terms of PA measures, the self-reported survey was used to identify the minutes per day and days per week of PA for three intensity levels (light, moderate, and vigorous) as well as transportation and recreation walking within and outside respondents' neighborhoods. Relevant survey items were adapted and/or modified from the International Physical Activity Questionnaire (IPAQ) to measure hours and minutes of MVPA on a typical day of the week which can also be translated to weekly measures of MVPA for comparisons to PA guidelines.

For objective measures, the ActiGraph GT3X+ accelerometer was used to capture the total minutes of PA by intensity levels (light, moderate, and vigorous). Additionally, participants were asked to wear a Qstarz BT Q1000 XT GPS unit to provide the location data for all outdoor PA and the speed of movement data to help more accurately detect walking activities (85, 86). In addition, a travel log was used to record the origin and destination, start and end times, the purpose of each trip, and PA type and duration at destinations on a small paper booklet. This information provided complementary data to help confirm the trip purpose and validate the data captured from the devices (87). The combination of the accelerometer and GPS data, supplemented by the information from the travel log, allowed for accurate measures of the PA outcome variables.

Participant training manuals for objective measures helped participants follow standard protocols for the use of devices and travel logs. A field manager delivered the devices with an instruction card and contact information and personally demonstrated how and when to wear the devices and charge the GPS unit. When completed, the devices were picked up by our field manager or dropped off at a designated location. The data from the devices were downloaded immediately after the devices were returned, and those who failed to provide minimally valid data (i.e., 4 days with 10+ h of valid data per day) were asked to re-wear the devices. Respondents were offered incentives (i.e., gift cards) for participation, including the chance to win more substantial gift cards through a raffle mechanism at the end of data collection.

For each participant, accelerometer data were processed using a bout of 7+ consecutive minutes with a two-minute tolerance. The total counts of bouts were averaged over the total wear time, and more than 90 consecutive minutes of zero activity counts were considered non-wear time. Standard/accepted activity count thresholds (e.g., 2,691–6,166 counts/min for moderate intensity and >6,166 counts/min for vigorous intensity based on recommendations from previous studies) (88) were used to determine the PA intensity level, and to estimate the total MVPA.

Spatial and temporal patterns of PA were captured by (a) the percentage of PA that takes place within the Mueller community or their comparison neighborhoods among the total PA, (b) the percentage of PA that is accounted for by walking, and (c) the frequency and distribution of PA episodes per day (as an indicator for the extent PA is integrated into daily routines).

One of the challenging prerequisites to obtain these measures was to detect PA types/modes. We used a machine learning approach (e.g., two-phase recognition, Tree-Structured Parzen Estimator, Random Forest) to detect target PA modes including walking, biking, driving, and sedentary behavior, and to estimate the hourly, daily, and weekly minutes of each mode within and outside each participant's neighborhood. The final training, validation, and test sets achieved an accuracy of 96.7% for the training data in detecting walking, and 77.3% for the test data in detecting walking, utilizing the data collected from the specific accelerometer and GPS devices and the travel log template that this study used. The variables generated from the machine-learning algorithms will be used with the detailed geospatial data generated from GIS, GPS, travel logs, and street audits to explore the spatial and temporal patterns of PA as described above.

#### Measurement of Demographic and Psychosocial Factors Using Survey Items

Demographic and psychosocial factors were captured through the survey. If participants needed assistance filling out the survey, the fieldworker provided such assistance. Control variables in the survey include demographic factors such as age, sex, race, ethnicity, education, marital status, and household composition; general self-assessed health (including number and type of co-morbid conditions); and quality of life (Table 2). The follow-up survey also assessed intermediate psychosocial outcomes/mediators such as attitudes toward PA (e.g., self-efficacy, beliefs about PA), and social influences (e.g., social support, neighborhood cohesion, perceived safety) (Table 2).

#### Measurement of Physical Environment Using GIS and Field Audits

Analyses of objectively measured neighborhood environments included the use of GIS software, ArcGIS, as well as field audits (89). GIS measures include *proximity measures* (distance from home to the closest destination) and *buffer measures* (characteristics within 0.5-mile of three types of buffers—airline, network, and sausage buffers—around each survey respondent's home). Proximity measures include distances to both *utilitarian* (grocery stores, restaurants, retail stores, banks, post offices, education, and community facilities, religious facilities, etc.) and *recreational* (parks, trails, gyms, etc.) destinations. Buffer measures include: (a) overall *land use characteristics* (percent of different land uses, number of utilitarian and recreational destinations, residential density, land use mix/diversity); (b*) street characteristics and walkability* (street connectivity, sidewalk completeness, marked crosswalks, bike lane, traffic signals and stop signs, and transit stop); (c) *safety* (crime and crash density, posted speed); (d) *natural environment* (tree canopy coverage, greenery coverage, water coverage, mean slope); and (5) *others* (socio-demographic characteristics, affordable housings, residential appraisal value, number of jobs, and construction permits).

#### Measurement of Weight, Height, and Body Composition

Our trained research staff also measured participants' weight and body composition using a portable Tanita Scale (TBF-400 Total Body Composition Analyzer) and height measure, which has been tested for validity/accuracy and has been used in several studies to obtain clinical measures of body composition (90–93). Height was measured by a portable stadiometer (HM-200P Portstad) (94, 95). These measures were collected during the research staff's home visits to deliver the objective measurement devices. Measurements were taken twice, and their mean values were used for the analysis.

### Analysis Plan

A standard data management protocol will be used, including detailed coding manuals and pre-processing protocols such as data cleaning, integration, reduction, and transformation, to ensure data quality and accuracy. Results of preliminary analyses will then be applied to determine appropriate statistical models to be applied.

Analyses of data will include descriptive statistics, graphical exploratory data analytic techniques to (a) describe distributions of the data, (b) identify outliers and missing values, and (c) check for the violation of assumptions necessary for the planned statistical methods. If statistical assumptions are severely violated, variables may be transformed, or analogous non-parametric tests may be used. Further analyses will include evaluating the population representativeness of the sample because of exclusions or dropouts while checking the comparability of treatment groups and the need for covariate adjustment using bivariate analyses. Statistical software for analyses will include but is not limited to SAS (version 9.4) (96), SPSS (97), STATA (98), R (99), R packages *lme4, mediation*, and *SpatStat*, for exploratory analysis, visualization of spatial-temporal point patterns data, patterns and mechanisms of missing data, and sensitivity analyses to assess the robustness of the analysis methods, including addressing any missing data problems (100).

For *Hypothesis 1A*, analyses will include paired *t*-tests and difference-in-differences methods to test pre-post changes and case-comparison differences in total MVPA per week. First, *t*-tests will be conducted using the pwr.t.test function of R software and follow Cohen's method (101). Required sample sizes were calculated by assuming two-tailed paired sample *t*-test and a 0.05 significance level to achieve the power of 0.8. We referred to the effect sizes in (a) our preliminary study (75) on self-reported PA among insufficiently active residents moving to Mueller from non-AFCs (effect size: 0.95), and (b) the effect size (0.50) from another study (84) of accelerometer-measured MVPA for residents from communities with different levels of walkability. The smaller effect size (0.50) was selected to reach a conservative estimate. The target sample sizes to detect pre-post differences in total MVPA per week in Hypothesis 1A using *t* tests are *34 pairs at first follow-up* for a power of 0.8. In addition, we will also use the difference-in-differences method to test the significance of the pre-post differences between the case and comparison participants. This method will help reduce the impact of uncaptured confounding factors and isolate the treatment (i.e., moving to Mueller in this case) effect (102, 103). For *Hypothesis 1B*, analyses will include repeated-measures linear mixed models to compare PA levels at 1st and 2nd follow-up with baseline levels. This method has the advantage of allowing an unequal number of repeated measurements over time. Analyses will also include the use of the *lme4* function in R software. We will use G^*^Power software to compute the conservative power estimates for a repeated measure ANOVA analysis with three repeated measures and two case and control groups. Assuming a 0.05 significance level, a small effect size of Cohen's *f* = 0.1, a moderate correlation of 0.6 among repeated measures, and an attrition rate of 20% at the 2nd follow-up, the target sample sizes for the smallest group to detect changes of PA levels for Hypothesis 1B are *82 pairs at 2nd follow-up* to reach a power of 0.8.

For *Hypothesis 1C*, analyses will include machine learning, artificial intelligence, and pattern recognition techniques to characterize activities with respect to the exact type of PA (walking, running, etc.) as well as for the classification of activity spaces and purposes (e.g., exercise or utilitarian) using a combination of location (GPS), time (GPS + accelerometer), and activity intensity information (accelerometer). These classification techniques are based on the individual patterns of each study participant and are learned automatically as the participants wear the devices over the study period. These classifications of activities within specific activity spaces provide the ability to independently assess the roles that each of the built and natural environmental factors play in promoting or hindering PA around the participant's home, workplace, and other activity spaces. They also help quantify the effects of these drivers on observed changes in levels of PA for both exercise/recreational and utilitarian purposes. These high-level, per-individual activity patterns can then be integrated with the static GIS data layers that characterize aspects of the built and natural environments and for utilization within spatial-temporal data analysis techniques. Spatial-temporal patterns of specific types of PA will then be examined using the *marked space-time Poisson point pattern analysis* (104). The observed locations and times of specific PA are treated as random points from a Poisson process and the corresponding types of PA are treated as marks. For each type of PA, the random space-time point of PA is assumed to follow a Poisson process with a space-time varying intensity, which we estimate by nonparametric intensity estimation approaches in the R package *SpatStat*. This point process model will allow for describing, visualizing, and evaluating spatial-temporal patterns of PA.

For *Hypothesis 2*, analyses will include hierarchical linear mixed models to describe longitudinal repeated measurements of PA in the presence of mediator effects (105). Variables of built and natural environmental factors and latent mediator variables of psychosocial factors are incorporated into linear mixed models as fixed effects and mediation effects, respectively, to investigate the direct and indirect associations between physical environmental explanatory variables and increased PA. We will also incorporate random effects into our model to account for dependence among repeated measures. Specifically, let *PA*_*i, t*_ denote the total PA levels measured on individual *i* at time point *t*, for *t* = 1, 2, 3. Let *BN*_*i, t*_ denote a vector of physical environmental explanatory variables, and *PS*_*i, t*_ denote a vector of psychosocial variables. Our linear mixed model consists of two model equations: *PA*_*i, t*_ = *X*_*i, t*_β_*BN*_ + *M*_*i, t*_β_*M*_ + *L*_*t*_ +ε_*i, t*_, *M*_*i, t*_ = *PS*_*i, t*_γ_*PS*_ + *BN*_*i, t*_γ_*BN*_ + η_*i, t*_, *where M*_*i, t*_are latent psychosocial mediators that are modeled as linear regressions with explanatory variables as measurements of psychosocial factors and built and environmental factors, *X*_*i, t*_ *is* a vector of covariates for fixed effects including physical environmental factors, temporal trend, and treatment effect, and *L*_*t*_ is modeled as a random effect to account for dependence between repeated measurements within the same participant.

Linear mixed models deal with unbalanced repeated measures due to attrition. We will use functions in R package *lme4* for fitting, analyzing, and testing longitudinal fixed and random effects in linear mixed-effects models, and use functions in R package *mediation* for the mediation analysis. Missing values, as needed, can be handled by following the multiple imputation approach by Schafer to improve efficiency and correct potential bias in parameter estimations (106).

## Discussion

### Summary of Major Changes to the Original Protocol

During the process of implementing this study, some adjustments were made to the initial study protocol due to the unique yet commonly occurring challenges in natural experiment studies (107). The procedures reported above reflect the final/actual protocol that was implemented, while this section summarizes major adjustments made to the original protocol. The lessons from this process represent typical challenges in natural experiments when researchers cannot randomly assign participants to treatment. As a result, research teams should be ready and able to implement alternative strategies as needed to ensure the success of the overall project. We hope the lessons learned from implementing this study can inform future planning efforts in similar community-based natural experimental studies in community settings.

The major challenge emerged from difficulties in recruitment as we needed to identify case participants before their move from various locations that were not known to the researchers in advance and the contact information of these target participants was not directly accessible. This challenge was further complicated by the fact the baseline assessment was limited to a fleeting time window as case participants, especially apartment residents, were often identified and recruited shortly before their move. In response to these challenges, several adjustments were made to our original protocol. First, we expanded our recruitment criteria to include not only those who were insufficiently active at baseline (as originally proposed) but also those meeting the PA guidelines, to increase the sample size for the analysis. Second, the difficulty and the corresponding delay in recruitment also made it necessary to reduce the total of four waves of follow-up assessments (originally planned at ~6, ~12, ~18, and ~24 months post-move) to two waves (at about 6–12 and 12–24 months post-move).

Furthermore, we had to employ additional recruitment sources such as advertisements on local media and referrals through the social network of local residents and leasing agents at apartment complexes, for which a modest amount of cash incentive was provided. In addition, like many other studies, our recruitment and follow-up challenges were amplified by the COVID-19 pandemic, which made face-to-face recruitment and objective assessments more challenging.

We will also face a challenge in population representativeness that will need to be addressed as a potential limitation. Although the population characteristics of Mueller and other Austin neighborhoods were similar at the early stage of our research, some differences have emerged over time. More up-to-date information from the 2019 American Community Survey (108) 5-year summary data showed that compared with the City of Austin, the Mueller population has a higher percentage of White (59.0% vs. 48.3% for Mueller vs. citywide mean), non-Hispanic, or Latino (79.6% vs. 66.1%), and residents with graduate or professional degree (36.2% vs. 19.4%). In terms of the household annual income, the Mueller population has a higher percentage of both high-income households (28.8% vs. 19.0% with $150,000+) and low-income households (19.2% vs. 15.1% with < $25,000). Covariate analyses can help mitigate these differences.

### Advantages of the Transdisciplinary Team

The transdisciplinary nature of our team was a major asset. We had members from urban planning, landscape architecture, architecture, public health (health behavior, health policy), exercise physiology, statistics, and computer sciences. The unique training and research experience gained from a diverse and transdisciplinary team allowed us to approach research questions that might not otherwise have been asked in a more siloed approach, and as such, this was a major strength. For example, the team engaged in the development of data collection protocols, surveys, planning for knowledge dissemination, engaging key stakeholders, and so forth, all informed collectively by the interactions of multiple disciplines, but in an integrative team approach that might not otherwise have been available. This approach also allowed us to go beyond the traditional boundaries of a field-specific approach, and to capture items that may not be common in a single field. We recommend taking multidisciplinary, interdisciplinary, and/or transdisciplinary approaches where possible, realizing these approaches are not identical (109). Given the value added in the current study and that the National Institutes of Health (NIH) (110) and others (111) have embraced this approach, we believe this was of significant value in the current study.

## Conclusions

This study's *longitudinal design and inclusion of case and matched comparison* participants allow us to better specify the impact of moving to an AFC on population-level behavior changes toward more physically active lifestyles. Moving into an AFC provides a proxy test for the health effects of innovative land-use policies that facilitate the development of AFCs. Using this timely opportunity for gaining longitudinal assessments for this natural experiment is of critical importance to advancing the status of knowledge on the intersection of health and place, as it relates to promoting PA as a means to enhance healthy living/aging by reducing the risk for chronic conditions such as cancer, diabetes, and heart disease. Not only do we expect more favorable health outcomes for AFC residents, but we also expect that this research may have positive effects on the community and private development investment in AFC environmental features.

## Ethics Statement

The studies involving human participants were reviewed and approved by Texas A&M University Institutional Review Board. The participants provided their written informed consent to participate in this study.

## Author Contributions

XZ, CL, and MO co-led the development of the Active Living Austin study protocol presented in this manuscript, with other co-authors providing assistance for their specific area of expertise. XZ led the writing of this manuscript. CL and MO oversaw the overall protocol and writing, all other co-authors helped with the writing and review. MO and ST assisted with the section about surveys. MX and JL aided with the section about objective measures of physical activity. CL, HL, and ZL helped with the description of environmental measures. TH, MX, and CL helped with the section about machine learning. EM and LS helped with the qualitative section of the study protocol. HS provided the description of the data analysis plan. All authors contributed to the article and approved the submitted version.

## Funding

This study is funded by the National Institutes of Health (Grant ID: R01CA197761).

## Conflict of Interest

The authors declare that the research was conducted in the absence of any commercial or financial relationships that could be construed as a potential conflict of interest.

## Publisher's Note

All claims expressed in this article are solely those of the authors and do not necessarily represent those of their affiliated organizations, or those of the publisher, the editors and the reviewers. Any product that may be evaluated in this article, or claim that may be made by its manufacturer, is not guaranteed or endorsed by the publisher.
